# Challenges and opportunities in understanding dementia and delirium in the acute hospital

**DOI:** 10.1371/journal.pmed.1002247

**Published:** 2017-03-14

**Authors:** Thomas A. Jackson, John R. F. Gladman, Rowan H. Harwood, Alasdair M. J. MacLullich, Elizabeth L. Sampson, Bart Sheehan, Daniel H. J. Davis

**Affiliations:** 1 Institute of Inflammation and Ageing, University of Birmingham, Birmingham, United Kingdom; 2 University Hospitals Birmingham NHS Foundation Trust, Birmingham, United Kingdom; 3 Division of Rehabilitation and Ageing, Queen’s Medical Centre, Nottingham, United Kingdom; 4 Nottingham University Hospitals NHS Trust, Nottingham, United Kingdom; 5 Edinburgh Delirium Research Group, University of Edinburgh, Edinburgh, United Kingdom; 6 Marie Curie Palliative Care Research Department, Division of Psychiatry, University College London, London, United Kingdom; 7 Psychological Medicine, Rehabilitation and Cardiac Division, John Radcliffe Hospital, Oxford, United Kingdom; 8 MRC Unit for Lifelong Health & Ageing, University College London, London, United Kingdom

## Abstract

In an Essay, Andrew Jackson and colleagues discuss challenges in the diagnosis and management of older people with dementia and delirium in acute hospitals.

**Summary points**

- Dementia in acute hospitals is common and associated with poor health outcomes.
- Dementia in acute hospitals is intricately linked with delirium, and the two should always be considered together when developing future policy.
- The decline in health and function after hospitalization among people with dementia may be influenced by discrete disease processes but also by the hospital environment and care itself.
- Opportunities for further research into the specific acute hospital management of dementia and its complications are many.

## What is the problem?

### Dementia in general hospitals

Dementia is very common in patients admitted to acute hospitals, affecting one in four patients, with 6% of people living with dementia being inpatients in acute hospitals at any given time [1,2]. Dementia is often unrecognised by doctors and other hospital staff and frequently complicated by delirium. Deficiencies in care have been highlighted by national audit and numerous reports [3].

“Intellectual failure” is recognised as one of the “geriatric giants.” Both delirium and dementia are disorders of cognitive function, are associated with adverse health outcomes, and are intricately linked [4]. Understanding how to assess, manage, and follow up older people with cognitive impairment in hospitals is vital to improving their care.

This essay discusses the clinical manifestation and complications of delirium and dementia in acute hospitals. Diagnosis of both conditions can be uncertain, and treatments are limited, but effective actions and management may improve outcomes. We also highlight areas for future research and suggest policy interventions to improve hospital care.

### Prevalence, presentation, and recognition

Estimates of the prevalence of dementia in hospitals vary across published studies [5] but range between 15% to 42% [6–10]. To put this in context, if two-thirds of hospital bed-days are in people over 65 years of age, then 25% of people in general hospitals will have dementia. However, published reports vary in ascertainment method and whether they distinguish between delirium and dementia.

People with dementia and cognitive impairment are hospitalised for many reasons, but typically in crises. Admission problems include immobility (73%), falls (64%), pain (54%), and breathlessness (23%) [11]. Patients in hospital with dementia are 4 to 7 years older, more likely to be women, and more likely to live in a care home than those without dementia [5]. Three-quarters of hospitalised patients with dementia have been defined as frail, compared to one-quarter of similar people without dementia [7].

Although dementia prevalence is high, the proportion undiagnosed or unrecognised by health care staff is approximately 56% (data in S1 Table) [6–8,12]. In older patients with delirium, only 36% of those with dementia had a recognised diagnosis [13]. Extrapolating these figures to a typical 500-bed general hospital suggests there would be at least 70 inpatients with unrecognised dementia at any one time.

### Severity

Hospitalised people with dementia typically have more advanced disease than those in the community. A Functional Assessment Staging Scale (FAST) stage of 6d (nearly mute, immobile, and incontinent) or above is present in 46% of hospitalised patients with dementia [14]. Three-quarters of patients with dementia in hospital had behavioural and psychological symptoms of dementia (BPSDs), and 43% had symptoms that challenged the staff involved [14]. These rates are much higher than would be expected in a general population with dementia in community settings. Patients with dementia in hospital also have a lower quality of life [15]. BPSDs in hospitals are often treated pharmacologically with antipsychotic drugs or benzodiazepines, which is associated with a nearly 3-fold increased risk of hospital mortality [16].

### Adverse outcomes

Dementia in general hospitals is associated with more inpatient adverse events, principally mortality, falls, and delirium [17], with increased costs of care [18]. Studies suggest a mortality rate of 31% at 6 months and 40% at 12 months, with a large increase in care-home residence at 12 months [19]; of the patients studied, 24% were new institutionalisations, and 42% were readmissions [20]. Even abnormal scores on single tests (either the Mini-Mental State examination or the Clock Drawing Test) were associated with mortality at 1 year (hazard ratio [HR] 2.9 [95% CI 1.3–6.4]) [21].

**Fig 1:**
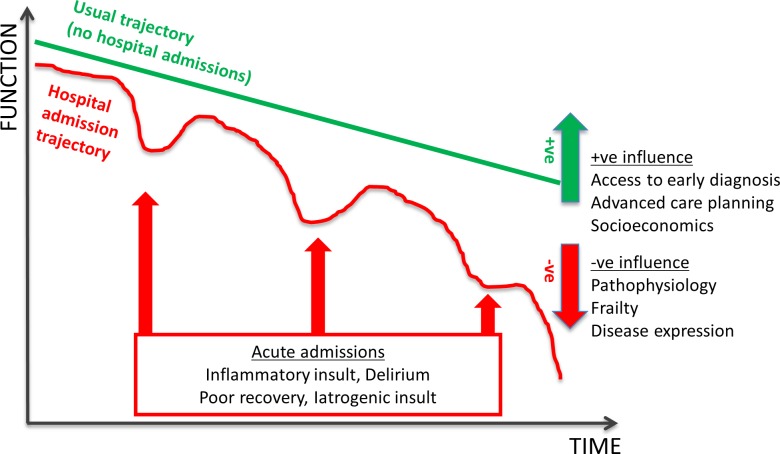
Schematic representation of dementia disease trajectory over time influenced by hospital admission. Dementia disease trajectories between a person with no hospital admissions (green line) and multiple hospital admissions (red line) are illustrated. The disease trajectory is negatively influenced by baseline frailty and disease expression. However, it may be positively tempered by early diagnosis, leading to better access to services, and advanced care planning. The “multiple hospital admissions” trajectory is further influenced by specific hospital interactions—importantly, delirium—but there are other effects from an acute inflammatory insult, subsequent recovery, and in-hospital iatrogenic insults.

The worst outcomes seen in people with dementia may be avoidable if they are due to poorer standards of care provision. Hospital staff can struggle to meet the complex care needs of people with dementia, often leading to a negative perception of such patients [22]. Iatrogenic and/or hospital environmental factors may lead to significant harm, and the inpatient care of people with dementia could be seen as a quality indicator for hospitals (as cited in the United Kingdom Francis report). However, biological factors also impact upon outcomes. It has been hypothesized that the interaction of an acute inflammatory event may accelerate functional and cognitive decline in this vulnerable population (as shown in Fig 1). Research to understand these mechanisms and to develop intervention strategies to minimise these outcomes is urgently needed. S2 Table summarises key studies presented above.

### Impact of delirium on older people in hospital with dementia

Delirium is an acute, severe neuropsychiatric syndrome seen mainly in older people in hospital and associated with increased morbidity and mortality [23]. Dementia is the strongest risk factor for developing delirium [24], with delirium superimposed on dementia accounting for 65% of delirium cases in hospital [25]. Delirium is associated with worsening of dementia and is a risk factor for subsequent dementia [26,27], with only 19% of people with delirium free from cognitive deficits 3 months later [13]. Those with dementia and delirium have the poorest outcomes [28]. In the context of the acute general hospital, dementia and delirium are intricately linked, and it is difficult to effectively recognise, investigate, manage, and suggest policy about one without the other, although the UK National Dementia Strategy only briefly mentioned delirium [29].

### Delirium Superimposed on Dementia (DSD)

The diagnostic challenge in an older person presenting with “confusion” is to disentangle whether they have delirium, dementia, or both. Persistent delirium is also possible [30]. Delirium in people with dementia is especially likely to go unrecognised [31]. There is a major need for better fundamental research to characterise these conditions biologically and clinically to improve care. However, although no simple diagnostic criteria exist, there are clinical processes that enable accurate diagnosis.

### Diagnosing delirium in people with dementia

Arousal and alertness are usually abnormal in delirium, but these domains are also increasingly affected in severe dementia. Diagnosing delirium in a person with dementia requires competence in cognitive testing, mental state examination, and informant questioning. Few screening tests have tried to detect delirium in the context of dementia; the Confusion Assessment Method (CAM) and the 4AT are examples [25,32].

There are four core challenges when diagnosing delirium in dementia. First, dementia with Lewy bodies (DLB) causes around 4% of all cases of dementia [33] and typically presents with a more rapid onset and with fluctuating degrees of cognitive impairments, attentional deficits, visual hallucinations, and paranoid delusions. This presentation may be similar to delirium, especially persistent delirium. Secondly, the impact of the hospital environment and sleep deprivation can lead to sleepiness by day, irritability, and behavioural “challenge” even in the absence of delirium. Thirdly, the progression of vascular dementia can deteriorate suddenly. Fourthly, BPSDs in dementia, typically including altered arousal, hallucinations, and agitation, are present in 75% of people with dementia in hospital [14]. However, given the seriousness of delirium, best practice is to assume delirium and manage as such until proven otherwise.

### Diagnosing dementia in people with delirium

By definition, manifestations of delirium follow an acute and fluctuating course; therefore, traditional tools to detect dementia by measuring cognitive deficits assumed to be stable are not useful. Many tools available to detect dementia in hospitals have not been validated in patients with delirium [34]. Obtaining information about baseline premorbid cognition from an informant is critical, but this requires attention and skill and is often badly done. The Informant Questionnaire of Cognitive Decline in the Elderly short form (IQCODE-SF) and the AD8: The Washington University Dementia Screening Test (AD8) have been validated to detect pre-existing dementia in older people with delirium [35]. Defining the duration of delirium is difficult, and current health services are rarely configured to review cognition after an acute episode.

## What is the solution?

### Management of people with dementia in hospital

Despite the clear need, little research is available on how best to provide care. The patient and carer experience of care is often negative, with deterioration in health, perceived poor care, and unrealistic expectations cited [36,37]. However, outcomes in dementia can be improved. For example, comprehensive geriatric assessment of patients with hip fracture and dementia leads to better functional mobility [38]. In-hospital fall prevention strategies can reduce falls, including in those with cognitive impairment [39]. Delirium prevention strategies have shown a reduction in delirium and falls [40]. Research specifically in delirium prevention for dementia populations is scarce [41]; however, it is reasonable to assume a general effect that extends to people with dementia.

Evidence to inform nutritional support [42] and specific therapy interventions related to discharge planning are lacking but may be crucial to provide patients with the best chance to be discharged home, as opposed to inappropriate institutionalisation. Engaging and listening to families is vital to ensure appropriate discharge planning at an early stage [43]. Families can also be enabled to recognize delirium in people with dementia, as well as to ensure recognition of its resolution or lack thereof, during the inpatient stay.

Joint units with geriatric medicine and psychiatry may reduce length of stay and readmissions [44]. One randomised controlled trial of a specialist unit for older patients admitted to hospital with confusion found no impact on length of stay or institutionalisation when compared with usual care but did show improvements in care interactions, carer satisfaction, and cost-effectiveness [45,46]. Efforts to improve care are hampered by the lack of specific treatments for delirium with dementia, either pharmacological or nonpharmacological.

### Managing undifferentiated cognitive impairment in hospital

The typical approach to manage cognitive impairment has been to attempt to diagnose delirium, dementia, both, or something else. There is a case for recognising these in-hospital conditions as a complex discrete syndrome, not least because some aspects of management are the same whatever the underlying diagnosis may be [47]. Undifferentiated cognitive impairment management includes treating patients as if they have delirium and possibly dementia. Unless the history is very clear, this should involve detailed characterisation of symptoms and impairments without necessarily giving a label, performed as part of comprehensive geriatric assessment. Therefore, management can be need driven, rather than diagnosis driven.

## What needs to happen next?

Assessing cognitive impairment, adverse events risk, and BPSDs should become routine. Delirium prevention, active management of underlying precipitants, and a patient safety approach to minimise harms are especially important [48]. Hospital care requires appropriate environmental adjustments (colour, light, visual interest, orientation, and furniture) and processes (person-centred care, attention to meals, activity, and sleep promotion), delivered by increased and upskilled staff. Proper attention should be given to legal aspects, especially around consent, and to risk enablement [49]. The recent interdisciplinary collaboration between the American Delirium Society and the American Nurses Association is an example of moving this evidence into practice [50]. Delirium and dementia patients will be slower to recover, and return to home living should be based on “adaptive” rather than “restorative” rehabilitation.

Dementia is a long-term condition for which the aim is to “live well.” Given that outcomes after hospitalisation are poor, an acute hospital admission should trigger a palliative needs assessment with discussions about goals and expectations of treatments, as part of a shared decision-making process. These discussions are time consuming and difficult in the face of uncertainty but reflect best practice.

There is a dearth of treatments for dementia and delirium and as yet no reliable and meaningful biomarkers to guide management. Evidence is lacking on how best to incorporate carers into hospital care as well as how to best train a fit-for-purpose workforce [51]. Assistive technology may in the future enhance dementia care [52], and further trials of specialized units are needed. However, any trials would need to be carefully designed with outcomes that are important to very physically and cognitively frail people, half of whom are in the last year of life.

### Conclusions

Despite challenges, the proactive diagnosis of dementia and delirium in hospitals is likely to improve patient experience and outcomes. Because cognitive impairment is so common in hospitals and impacts so substantially on long-term outcomes, there is a pressing need for (1) joined-up care to alter a trajectory of decline and (2) more research to improve diagnostics and management, whatever the specific underlying diagnosis.

## Supporting information

S1 Table Proportion of dementia unrecognised in hospital cohorts. (DOCX)

S2 Table Characteristics and key findings from a systematic review and two major cohorts describing dementia and cognitive impairment in general hospitals. (DOCX)

## Abbreviations

AD8: The AD8: The Washington University Dementia Screening Test
BPSD: behavioural and psychiatric symptoms of dementia
CAM: Confusion Assessment Method
DLB: dementia with Lewy bodies
DSD: delirium superimposed on dementia
FAST: Functional Assessment Staging Scale
HR: hazard ratio
IQCODE-SF: the Informant Questionnaire of Cognitive Decline in the Elderly short form
