# Supplementary material for: Challenges and opportunities in understanding dementia and delirium in the acute hospital
Source: PLoS Med. 2017 Mar 14;14(3):e1002247. doi: 10.1371/journal.pmed.1002247 (PMC5349650; doi:10.1371/journal.pmed.1002247)
Supplement: S1 Table — (DOCX) [file pmed.1002247.s001.docx]

**S1 Table: Proportion of dementia unrecognised in hospital cohorts**

| Study | Dementia ascertainment | Previously diagnosed | | % unrecognised |
| --- | --- | --- | --- | --- |
| General hospital | | | | |
| Briggs 2016, Timmons 2015  [1, 2] General hospital, Ireland | DSM-IV | 53/149 | 64% | |
| Travers 2013  [3] General hospital, Australia | Expert Dx | 43/102 | 58% | |
| Sampson 2009  [4] Medical admissions, UK | Expert Dx | 130/262 | 49% | |
| Laurila 2004  [5] Geriatric Medicine, Finland | Expert Dx | 31/77 | 60% | |
| Pooled data from above studies |  | 257/590 | 56%  (95% CI ±4.01) | |
| Delirium | | | | |
| Jackson 2016  [6] Pnts with delirium, UK | DSM-IV | 30/47 | | 36% |
| Ryan 2013  [7] Ptns with delirium | IQCODE + expert Dx | 5/28 | | 82% |
| Specific populations | | | | |
| Partridge 2014  [8] Vasc surgery, UK | MOCA <24/30 | 6/77 | | 88% |

Data expressed as n/N where N is the number of people identified with dementia and n is the number of those in whom the diagnosis was recognised by healthcare professionals.

% unrecognised is the proportion of people with dementia in whom the dementia was not recognised by healthcare professionals, (N-n)/N.

DSM-IV, Diagnostic and Statistical Manual of Mental Disorders fourth edition; UK, United Kingdom; Expert Dx, diagnosis ascertained after assessment by specialist in either psychiatry, neurology or geriatric medicine; IQCODE, Informant Questionnaire of Cognitive Decline in the Elderly; MOCA,

References:

1. Briggs R, Dyer A, Nabeel S, Collins R, Doherty J, Coughlan T, et al. Dementia in the acute hospital: the prevalence and clinical outcomes of acutely unwell patients with dementia. QJM. 2016.

2. Timmons S, Manning E, Barrett A, Brady NM, Browne V, O’Shea E, et al. Dementia in older people admitted to hospital: a regional multi-hospital observational study of prevalence, associations and case recognition. Age Ageing. 2015;44(6):993-9.

3. Travers C, Byrne G, Pachana N, Klein K, Gray L. Prospective observational study of dementia and delirium in the acute hospital setting. Intern Med J. 2013;43(3):262-9.

4. Sampson EL, Blanchard MR, Jones L, Tookman A, King M. Dementia in the acute hospital: prospective cohort study of prevalence and mortality. Br J Psychiatry. 2009;195(1):61-6.

5. Laurila JV, Pitkala KH, Strandberg TE, Tilvis RS. Detection and documentation of dementia and delirium in acute geriatric wards. Gen Hosp Psychiatry. 2004;26(1):31-5.

6. Jackson TA, MacLullich AM, Gladman JR, Lord JM, Sheehan B. Undiagnosed long-term cognitive impairment in acutely hospitalised older medical patients with delirium: a prospective cohort study. Age Ageing. 2016;45(4):493-9.

7. Ryan DJ, O'Regan NA, Caoimh RO, Clare J, O'Connor M, Leonard M, et al. Delirium in an adult acute hospital population: predictors, prevalence and detection. BMJ open. 2013;3(1).

8. Partridge JS, Dhesi JK, Cross JD, Lo JW, Taylor PR, Bell R, et al. The prevalence and impact of undiagnosed cognitive impairment in older vascular surgical patients. J Vasc Surg. 2014;60(4):1002-11.e3.

9. Mukadam N, Sampson EL. A systematic review of the prevalence, associations and outcomes of dementia in older general hospital inpatients. Int Psychogeriatr. 2011;23(3):344-55.

10. Sampson EL, White N, Lord K, Leurent B, Vickerstaff V, Scott S, et al. Pain, agitation, and behavioural problems in people with dementia admitted to general hospital wards: a longitudinal cohort study. Pain. 2015;156(4):675-83.

11. Sampson EL, White N, Leurent B, Scott S, Lord K, Round J, et al. Behavioural and psychiatric symptoms in people with dementia admitted to the acute hospital: prospective cohort study. Br J Psychiatry. 2014;205(3):189-96.

12. Glover A, Bradshaw LE, Watson N, Laithwaite E, Goldberg SE, Whittamore KH, et al. Diagnoses, problems and healthcare interventions amongst older people with an unscheduled hospital admission who have concurrent mental health problems: a prevalence study. BMC geriatrics. 2014;14:43.

13. Bradshaw LE, Goldberg SE, Lewis SA, Whittamore K, Gladman JR, Jones RG, et al. Six-month outcomes following an emergency hospital admission for older adults with co-morbid mental health problems indicate complexity of care needs. Age Ageing. 2013;42(5):582-8.

14. Whittamore KH, Goldberg SE, Gladman JR, Bradshaw LE, Jones RG, Harwood RH. The diagnosis, prevalence and outcome of delirium in a cohort of older people with mental health problems on general hospital wards. Int J Geriatr Psychiatry. 2014;29(1):32-40.
