# Supplementary material for: Challenges and opportunities in understanding dementia and delirium in the acute hospital
Source: PLoS Med. 2017 Mar 14;14(3):e1002247. doi: 10.1371/journal.pmed.1002247 (PMC5349650; doi:10.1371/journal.pmed.1002247)
Supplement: S2 Table — (DOCX) [file pmed.1002247.s002.docx]

**S2 Table: Characteristics and key findings from a systematic review and two major cohorts describing dementia and cognitive impairment in general hospital**

| Study | Population | Prevalence | Severity | In hospital associations | Outcome |
| --- | --- | --- | --- | --- | --- |
| Systematic review [9] | 14 studies >55 years General hospital medical and surgical inpatients Excluding hip fracture patients | 2.8% - 63% (14 studies) 25.1%-43.3% (3 studies using DSM-IV criteria) | Not described | Patients with dementia were older, female and from a nursing home (6 studies) | Increased length of stay (3 studies) Institutionalisation (2 studies)  increased delirium (1 study) |
| North London cohort [4, 10, 11] | >70 years old Unplanned acute admissions or medical unit Excluding surgical admissions N=617 83.0 yrs., 59% female | 42% with DSM-IV dementia 21.1% recognised diagnosis | 46% Functional assessment staging test (FAST) stage of >6d 75% BPSD 45% with mod/severe BPSD | Dementia has 2.18 RR of adverse event in hospital More likely to be admitted from care home More likely to have pressure sores | HR 2.09 for death during index admission 12 month outcomes: median survival 1.1 yrs. vs 2.7 years in those with dementia HR 1.66 for mortality (unadjusted) |
| Nottingham cohort [12-14] | >70 years old General medical and trauma orthopaedics admissions 1,004 screened, N=250 84.0 yrs., 66% female | 50% with cognitive impairment Half with those with a recognised diagnosis | 14% with delusions 20% with hallucinations 17% with agitation/aggression 38% with apathy | Cognitive impairment associated with being older, from a care home, with increased incontinence and greater functional dependence | 180 day outcomes: 31% died, 42% readmitted, 31% survived without being readmitted or moving to a care home |

DSM-IV, Diagnostic and Statistical Manual of Mental Disorders fourth edition; BPSD, behavioural and psychiatric symptoms of dementia.

References:

1. Briggs R, Dyer A, Nabeel S, Collins R, Doherty J, Coughlan T, et al. Dementia in the acute hospital: the prevalence and clinical outcomes of acutely unwell patients with dementia. QJM. 2016.

2. Timmons S, Manning E, Barrett A, Brady NM, Browne V, O’Shea E, et al. Dementia in older people admitted to hospital: a regional multi-hospital observational study of prevalence, associations and case recognition. Age Ageing. 2015;44(6):993-9.

3. Travers C, Byrne G, Pachana N, Klein K, Gray L. Prospective observational study of dementia and delirium in the acute hospital setting. Intern Med J. 2013;43(3):262-9.

4. Sampson EL, Blanchard MR, Jones L, Tookman A, King M. Dementia in the acute hospital: prospective cohort study of prevalence and mortality. Br J Psychiatry. 2009;195(1):61-6.

5. Laurila JV, Pitkala KH, Strandberg TE, Tilvis RS. Detection and documentation of dementia and delirium in acute geriatric wards. Gen Hosp Psychiatry. 2004;26(1):31-5.

6. Jackson TA, MacLullich AM, Gladman JR, Lord JM, Sheehan B. Undiagnosed long-term cognitive impairment in acutely hospitalised older medical patients with delirium: a prospective cohort study. Age Ageing. 2016;45(4):493-9.

7. Ryan DJ, O'Regan NA, Caoimh RO, Clare J, O'Connor M, Leonard M, et al. Delirium in an adult acute hospital population: predictors, prevalence and detection. BMJ open. 2013;3(1).

8. Partridge JS, Dhesi JK, Cross JD, Lo JW, Taylor PR, Bell R, et al. The prevalence and impact of undiagnosed cognitive impairment in older vascular surgical patients. J Vasc Surg. 2014;60(4):1002-11.e3.

9. Mukadam N, Sampson EL. A systematic review of the prevalence, associations and outcomes of dementia in older general hospital inpatients. Int Psychogeriatr. 2011;23(3):344-55.

10. Sampson EL, White N, Lord K, Leurent B, Vickerstaff V, Scott S, et al. Pain, agitation, and behavioural problems in people with dementia admitted to general hospital wards: a longitudinal cohort study. Pain. 2015;156(4):675-83.

11. Sampson EL, White N, Leurent B, Scott S, Lord K, Round J, et al. Behavioural and psychiatric symptoms in people with dementia admitted to the acute hospital: prospective cohort study. Br J Psychiatry. 2014;205(3):189-96.

12. Glover A, Bradshaw LE, Watson N, Laithwaite E, Goldberg SE, Whittamore KH, et al. Diagnoses, problems and healthcare interventions amongst older people with an unscheduled hospital admission who have concurrent mental health problems: a prevalence study. BMC geriatrics. 2014;14:43.

13. Bradshaw LE, Goldberg SE, Lewis SA, Whittamore K, Gladman JR, Jones RG, et al. Six-month outcomes following an emergency hospital admission for older adults with co-morbid mental health problems indicate complexity of care needs. Age Ageing. 2013;42(5):582-8.

14. Whittamore KH, Goldberg SE, Gladman JR, Bradshaw LE, Jones RG, Harwood RH. The diagnosis, prevalence and outcome of delirium in a cohort of older people with mental health problems on general hospital wards. Int J Geriatr Psychiatry. 2014;29(1):32-40.
